# Welfare traits of *Bos indicus* cattle castrated immunologically and fed beta-adrenergic agonists

**DOI:** 10.5713/ajas.19.0986

**Published:** 2020-04-12

**Authors:** Luciane Silva Martello, Daniel Silva Antonelo, Nara Regina Brandão Cônsolo, Verônica Madeira Pacheco, João Alberto Negrão, Alessandra Fernandes Rosa, Paulo Roberto Leme, Rafael Vieira Sousa, Saulo da Luz Silva

**Affiliations:** 1 College of Animal Science and Food Engineering, University of São Paulo, Pirassununga 13635900, Brazil

**Keywords:** *Bos indicus* Temperament, Cattle Behaviour, Infrared Thermography, Ractopamine Hydrochloride, Zilpaterol Hydrochloride

## Abstract

**Objective:**

This work was carried out to evaluate the effects of zilpaterol hydrochloride (ZH) and ractopamine hydrochloride (RH) combined with immunocastration on the welfare traits of feedlot Nellore cattle.

**Methods:**

Ninety-six Nellore males (average body weight [BW] = 409±50 kg; average 20 mo of age) were divided into two groups according to BW; half of the animals in each group received two doses of an immunocastration (ImC) vaccine in a 30 day interval, and the other half did not receive the vaccine (NoC). Afterward, the animals were housed and fed a common diet for 70 days. Then, they were split into three groups and fed one of the following diets for 30 additional days: control (CO) diet, with no β-AA; ZH diet, containing 80 mg/d ZH; and RH diet, containing 300 mg/d RH. Welfare traits were assessed by monitoring body surface temperature using infrared thermography (IRT) and plasma cortisol and temperament measurements.

**Results:**

There was no interaction between sexual condition and diet for any trait. The ImC and NoC groups did not differ in rectal and ocular temperatures. The ImC animals had higher flight speeds (p = 0.022) and tended to have higher cortisol levels (p = 0.059) than the NoC animals. Animals fed ZH and RH did not differ in cortisol levels, respiratory rate, rectal temperature, temperature measured by IRT, or temperament behaviour.

**Conclusion:**

The ImC animals showed a less stable temperament during handling practices than NoC, whereas ZH and RH supplementation had no adverse effects on animal welfare.

## INTRODUCTION

The use of feed additives in the finishing phase on the feedlot is a common practice used by the beef industry to improve animal performance, meat quality and profitability. Among these additives, beta-adrenergic agonists (β-AAs), such as zilpaterol hydrochloride (ZH) and ractopamine hydrochloride (RH), which are synthetic and nonhormonal compounds with a powerful anabolic effect on skeletal muscle, have been used to improve animal performance and lean meat production [1–3]. However, recently, the use of ZH was associated with the incidence of cattle suffering from stress and lameness after transportation to slaughter plants, which led to the suspension of ZH use for beef cattle operations in some countries. Despite this, the detrimental effects of β-AA supplementation on animal welfare are still unclear and inconclusive [4].

Castration is another practice that has been widely used in the beef industry because it decreases animal aggressiveness and sexual behaviour and improves fat deposition and carcass grading [5]. Nonetheless, this practice may be painful, stressful, and could negatively affect animal performance. Moreover, the increasing concern about animal welfare, mainly from those concerned about how some management practices can affect animal welfare, has driven the search for alternatives to some common practices used for animal production. In this sense, active immunization against GnRH, also called immunocastration, has been studied, with greater emphasis on its effect on performance and meat quality traits in beef cattle.

Nevertheless, to our knowledge, there have been no studies concerning the effects of β-AA supplementation and immunocastration on the welfare-related traits of *Bos indicus* cattle. Information about how these factors, independently or in combination, can affect production systems would help to better understand how they can be used to increase meat production and animal welfare. Therefore, this work was carried out to evaluate the effect of β-AA and immunocastration on the welfare traits of feedlot finishing *Bos indicus* (Nellore) cattle by monitoring body surface temperature using infrared thermography (IRT), plasma cortisol and temperament measurements.

## MATERIALS AND METHODS

This work was part of a larger project where performance, carcass traits and meat quality were reported by Antonelo et al [1], Brigida et al [6] and Mazon et al [7], respectively.

Ninety-six Nellore (*Bos indicus*) non-castrated males (409± 50 kg body weight [BW]; 20 mo old) were individually identified with ear tags, weighed, and divided into two groups according to BW (heavy and light). Thirty days before the beginning of the trial, half of each group received one dose of immunocastration vaccine (ImC; Bopriva, Zoetis Ltd., Campinas, SP, Brazil), whereas the other half did not receive the vaccine (non-castrated; NoC). A second dose of immunocastration vaccine was administered 30 days later. During this period (30 days), all the animals were kept together under pasture conditions.

After the second dose of immunocastration vaccine, the animals were moved to the feedlot facilities. One group (n = 48), composed of 24 ImC and 24 NoC animals, was distributed in four pens (12 animals/pen) equipped with electronic gates (American Calan Inc., Northwood, NH, USA). Each pen had only animals of the same sexual condition to avoid aggressive behaviour problems. Therefore, there were two pens with ImC animals and two with NoC animals. The second group (n = 48) was distributed in individual pens. Both facilities had covered feed bunks, concrete floors and automatic waterers.

Detailed information about diets, feeding and management practices can be found in Antonelo et al [1]. Briefly, all animals were fed a mixed common diet containing 76% concentrate and 24% roughage for 70 days. After that, animals were divided into three groups and assigned one of the following diets: control (CO), with no β-AA; ZH group, containing 80 mg/d zilpaterol hydrochloride (Zilmax, Merck Animal Health, Summit, NJ, USA); and RH group, containing 300 mg/d ractopamine hydrochloride (Optaflexx, Elanco Animal Health, Greenfield, IN, USA). The β-AA group was fed for 30 days (from day 70 to 100). Animals were slaughtered at day 103 because of the ZH withdrawal period (3 days before slaughter).

On days 70, 84, and 101 of feeding, the animals were weighed, and rectal temperature (RT), IRT, chute score (CS) and flight speed (FS) data were collected. Blood samples were collected for all animals at the beginning (day 0) and at slaughter, post-sticking at the abattoir (day 103), to measure plasma cortisol levels. Respiratory frequency was evaluated during 17 nonconsecutive days from day 70 to day 100 of feeding.

Blood samples were collected by jugular venipuncture, resulting in the collection of 4 mL of blood in BD Vacutainer tubes containing 158 USP sodium heparin (Becton Drive, Franklin Lakes, NJ, USA). These tubes were then inverted and placed on ice until centrifugation at 2,000×*g* for 20 min at 4°C. Plasma was separated and stored at −20°C until the analysis of cortisol levels. The plasma cortisol was analysed using a commercial kit (AccU-Bind – ELISA Microwells, Monobind, Lake Forest, CA, USA), and the levels were analysed by enzyme-linked immunosorbent assay, which measures the concentration by reading the absorbance of the unknown sample compared to the standard curve obtained from known concentrations (standard sample). The intra- and inter-assay coefficients of variation were 4.1% and 5.4%, respectively.

Due to the difficulty of individually measuring the respiration rate (RR) of all animals, eight animals per sexual condition×diet combination (n = 48 animals) were chosen to measure the RR. The RR measurements were performed with animals in their pens during the morning, starting at 7 am (before the RT and IRT measures), by counting flank movements three times for 15 s, and the average was used to calculate movements per minute (mov/min). The RT of all animals (n = 96) was measured manually using a digital clinical thermometer (VMDT01; Viomed, Yangzhou, Jiangzhou, China), which was inserted into each animal’s rectum while the animal was restrained in the squeeze chute, simultaneously with IRT measures.

**Figure 1:**
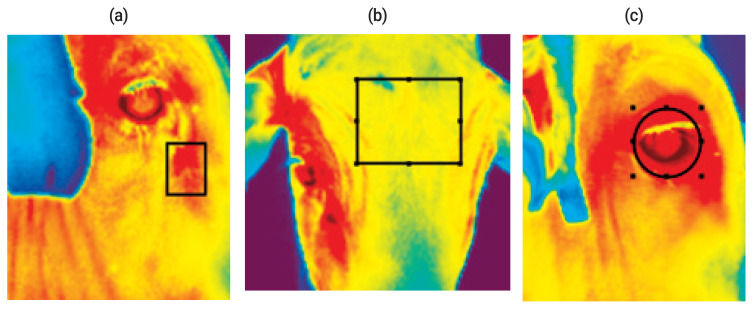
Infrared thermography images showing the sub-area of the body where the images were obtained: (a) cheek, (b) front, (c) ocular area.

Infrared thermography was performed using an infrared camera (TESTO 875-2i, Maharashtra, India) at the same time as the collection of RT and RR measurements. The emissivity value used was 0.98, which is the recommended value of the camera manufacturer for biological tissues. IRT images of the following three body locations of each animal were taken: cheek, front and ocular area. The images were interpreted using TESTO IRSoft (TESTO, India) software, considering the temperature of a specific point of each photographed body location (Figure 1). All IRT images were taken approximately 1.0 m from the animal.

The CS was evaluated by an observer who monitored the animals restrained in the squeeze chute and was determined using a note scale (Table 1) based on the observation of each animal restrained in the squeeze chute [8]. FS (m/s) was measured using pairs of photoelectric cells connected to an electronic timer (Duboi, Inc., Campo Grande, MS, Brazil) to determine the time taken for an animal to traverse a fixed distance of 5.5 m after exiting the squeeze chute, as previously described by Burrow et al [9].

To monitor the environmental conditions, a data logger (HOBO U12, ONSET, Bourne, MA, USA) was installed at the centre of the pens 2 m above the floor, which was approximately the level of the animals’ heads. Dry bulb temperature and relative humidity were automatically recorded at hourly intervals, 24 h a day, over the entire experimental period.

The effects of treatments on the response variables were evaluated in a randomized complete block design with a 2×3 factorial arrangement, considering sexual condition (NoC and ImC), diet (CO, RH, and ZH) and their interaction as fixed effects. The RT, RR, IRT, CS and FS were analysed as repeated measurements. For repeated measurements, covariance structures were modelled, and the best-fitted variance component was adopted. All analyses were carried out using the MIXED procedure of SAS (SAS Institute Inc., Cary, NC, USA). The animal was considered an experimental unit. When significant effects of main factors were observed, means were obtained using the LSMEANS option and compared by Student’s t test. Differences were declared significant when p≤0.05, whereas trends were declared when 0.05<p< 0.10.

## RESULTS

There was no interaction between immunocastration and β-AA for any trait evaluated. The descriptive statistics of individual measures are described in Table 2.

The initial cortisol levels were lower (3.9 μg/dL) than those observed at slaughter for both sexual conditions (5.3 μg/dL; p = 0.002; Table 2). There was no effect of sexual condition on cortisol levels measured at the beginning of the feedlot (Table 3); however, at slaughter a trend (p = 0.059) of higher cortisol concentration was observed in the ImC group than the NoC group.

The ImC animals had higher FS than the NoC animals (p = 0.023); however, no differences between sexual conditions were observed for CS. The ImC group tended to have higher RT (p = 0.083) and temperature of the ocular area (p = 0.059), as measured by IRT, than the NoC group. No differences between sexual conditions were observed for RR or IRT measured at the front and cheek.

ββ-AA did not affect cortisol levels either at the beginning or at slaughter. Similarly, β-AA had no effect on the temperament (CS and FS) or physiological traits such as RR, RT, and temperatures measured by IRT (Table 4).

## DISCUSSION

There has been increasing concern regarding the welfare issues associated with animal production [4,10,11], as some management practices can affect animal welfare with consequences on meat quality. Behavioural modification is the first reaction of animals to stress, followed by the activation of the central nervous system by the hypothalamic-pituitary-adrenal axis and the release of substances such as adrenaline and cortisol into the bloodstream. Therefore, blood cortisol levels can be used to quantify the stress response associated with handling [12]. In this study, cortisol levels were greater at slaughter than those measured at the beginning of the feedlot, which was expected because pre-slaughter handling is a stressful operation and results in increased cortisol levels [13,14].

ImC and NoC individuals showed no differences in cortisol levels at the beginning of feedlot; however, ImC tended (p = 0.059) to have higher levels at slaughter. Few studies have evaluated stress responses through cortisol levels between non-castrated and immunocastrated cattle. Andreo et al [15] and Bolado-Sarabia et al [16] also reported higher blood cortisol levels at slaughter for ImC males than for NoC (Nellore and Holstein breeds, respectively); however, the authors did not suggest any reason for the higher cortisol levels in the ImC animals. Although the focus of the present study is not to evaluate surgically castrated animals, it is interesting to mention what some studies have observed in this animal category in relation to cortisol levels. Studies comparing NoC with surgically castrated cattle reported lower cortisol levels in NoC [17–19]. The reason for the higher cortisol in castrated animals could be related to the altered steroid balance, due the trend of decreased adrenal sensitivity in animals with higher testosterone levels (NoC), reflecting a diminished cortisol response to adrenocorticotrophic hormone [20]. Studies in rodents [21] reported a suppressive effect of testosterone on stimulated hypothalamic-pituitary-adrenal axis activity, suggesting that testosterone suppresses stimulated cortisol secretion. In the present study, the ImC group had lower levels of testosterone [1], which could explain the higher cortisol levels. However, as there is not enough information available about cortisol patterns in ImC animals, it is difficult to say whether the response of the highest cortisol in ImC animals reflects a situation related to hormonal physiological adjustments related to testosterone levels or a condition of poorer welfare. Therefore, further studies evaluating the effects of immunocastration on serum hormones are needed to better elucidate its physiological effects.

The IRT is a fast, accurate and easy method of measuring animal body surface temperature [22–24]. The temperature of the animal’s skin may change according to variations in blood flow, which may be altered by the process of vasoconstriction or arterial vasodilation due to a stressful situation such as acute pain [25] or physical activities [26]. Physical activity can increase body temperature, leading to vasodilation and consequently increasing the surface temperature, contributing to heat loss in animals. To the best of our knowledge, no studies have evaluated the effects of immunocastration on animal body temperature. However, since immunocastration has been reported to reduce physical activity [27], due to the decrease in sexual and aggressive behaviour [5,11, 16], lower body temperatures were expected in the ImC group, which was not observed in this study, suggesting that both groups did not experience a stress situation during the trial.

In this study, the CS was not affected by sexual condition; however, ImC males had higher FS than NoC males. The higher FS observed in the ImC group was unexpected since NoC males are reported to have more sexual and aggressive behaviour. The CS test is used to measure the reaction of an animal when it is physically restricted in a shuttle, while the FS has been used to evaluate the fear response of animals when handled by humans [9,28]. Sant’Anna et al [8] observed a low phenotypic association between FS and CS, indicating that some individuals may have high values of FS without demonstrating any movement or agitation in the squeeze chute (low CS). In addition, *Bos indicus* cattle have different behavioural expressions of fear when entering the squeeze chute, exhibiting agitation by trying to escape or by freezing, which could explain the divergent results observed for CS and FS in the present study. Based on FS results, as well as the CS, IRT, RR, and TR results, it is possible to speculate that the higher FS observed for ImC reflects a less stable temperament for these animals when subjected to a stress situation such as handle in a squeeze.

The effects of using β-AAs, mainly ZH and RH, to improve the growth performance, carcass and meat yield of feedlot finishing cattle have been extensively studied in past decades and are well documented. However, few studies are available on the effects of β-AA on welfare-related traits. In the present study, animals fed either ZH or RH did not differ in blood cortisol levels, body temperatures measured by IRT, RT values, or RR values from the CO group. In contrast to this study, Boyd et al [4] reported that ZH-fed steers had higher RR and tended to have higher painting score values than control animals, although the ZH group had lower body temperatures. The authors commented that whether this increase in RR is due to a greater amount of heat load on the animal due the increased muscle mass or an unobserved biological effect of increased metabolism due to feeding ZH was not known. Hales et al [29] evaluated the effects of feeding ZH on heat stress in feedlot cattle and observed that even though the slopes for RR changed under different environmental conditions, no differences across dietary treatments were found, suggesting that feeding ZH for 21 d during the finishing period did not affect stress responses.

The assessment of the effects of β-AA on cattle should take into account the differences among species, breeds, sexual condition, environments, and days of β-AA feeding used in those studies. In this sense, notably, the present study was conducted under similar environments and ZH doses (80 mg/steer) used by Boyd et al [4] and Hales et al [30] but with differences in feeding period (21 d vs 30 d). These results suggested that the ZH feeding period could be increased for Nellore cattle without negative effects on animal welfare.

There are limited data regarding the effect of feeding β-AA feeding on cattle mobility. Baszczak et al [10] also found no effects of RH supplementation (200 mg/steer) on CS or FS in Brahman, British, and Continental cattle during a 28-d study period. These results are consistent with the findings of the present study, where no treatment effects were observed for CS or FS.

Based on these results, it is possible to suggest that feeding β-AA had no effect on animal stress and behavioural traits, since no differences were observed in cortisol levels, RT and RR values, ocular area temperatures, CS and FS values.

In general, it is possible to conclude that immunocastration induces a less stable animal temperament during handling practices, while feeding β-AA (ZH or RH) to Bos indicus cattle has no adverse effects on animal stress and behaviour traits.

## Figures and Tables

**Table 1:** Subjective score guidelines

Scores		Description
1	Nonreactive animal	Calm, idle and inattentive
2	Little reactive animal	Slightly restless, watchful
3	Reactive animal	Watchful with constant movement shaking of the chute
4	Very reactive animal	Violent and continuous struggling

**Table 2:** Descriptive statistics of traits evaluated and environmental conditions

Traits	Mean	SEM	Minimum	Maximum
Cortisol (μg/dL)				
day 0^1)^	3.9	2.08	0.25	9.68
day 103^2)^	5.3	3.12	0.93	18.59
Respiration rate (movements/min)	39.1	8.82	8.0	64.0
Rectal temperature (°C)	38.9	0.71	36.0	41.8
Infrared thermography (°C)				
Front	31.3	2.50	20.6	40.3
Cheek	32.6	1.61	25.2	38.2
Ocular area	33.3	1.35	27.5	37.4
Rib	33.1	1.86	26.8	39.1
Flank	32.7	2.42	25.2	42.4
Flight speed (m/s)	2.1	0.88	0.18	5.15
Chute score	2.0	0.96	1.0	4.0
Dry bulb temperature (°C)	27.2	5.24	12.2	36.3
Relative humidity (%)	62.7	19.12	23.0	94.3

SEM, standard error of the mean.

1) Blood sampled at the beginning of the feedlot period on day 0.

2) Blood sampled at slaughter on day 103.

**Table 3:** Effects of immunocastration on cortisol, respiration rate, body temperature, and temperament scores

Trait	Sex condition^1)^		SEM	p-value^*^

	NoC	ImC		
Cortisol (μg/dL)				
day 0^2)^	3.9	3.9	0.31	0.978
day 103^3)^	4.6	6.1	0.56	0.059
RR (movements/min)	25	25	0.58	0.775
Rectal temperature (°C)	38.7	38.8	0.48	0.083
Infrared thermography (°C)				
Front	31.9	31.7	0.23	0.616
Cheek	33.1	33.0	0.14	0.612
Ocular area	36.4	36.6	0.07	0.059
Chute score	1.9	2.0	0.07	0.978
Flight speed (m/s)	0.68	0.55	0.04	0.023

SEM, standard error of the mean; RR, respiration rate.

1) NoC, non-castrated males; ImC, immunocastrated animals (Bopriva, Zoetis Ltda, Campinas, SP, Brazil).

2) Blood sampled at the beginning of the feedlot period on day 0.

3) Blood sampled at slaughter on day 103.

* Means differ when p<0.05.

**Table 4:** Effects of feeding beta-adrenergic agonists (β-AA) on cortisol, respiration rate (RR), body temperature, and temperament scores

Trait	Diet^1)^			SEM	p-value

	CO	RH	ZH		
Cortisol (μg/dL)					
day 0^2)^	3.42	3.91	4.38	0.38	0.231
day 103^3)^	5.44	5.18	5.38	0.68	0.963
RR (movements/min)	25	24	25	0.71	0.848
Rectal temperature (°C)	38.8	38.8	38.7	0.05	0.694
Infrared thermography (°C)					
Front	31.8	31.8	31.7	0.28	0.989
Cheek	32.9	33.1	33.1	0.17	0.730
Ocular area	36.4	36.5	36.5	0.08	0.876
Chute score	2.1	1.9	1.9	0.09	0.354
Flight speed (m/s)	0.55	0.61	0.66	0.04	0.276

1) CO, basal diet with no β-AA; RH, ractopamine hydrochloride (300 mg/animal/d; Optaflexx, Elanco Animal Health, Greenfield, IN, USA); ZH, zilpaterol hydrochloride (80 mg/animal/d; MSD Animal Health, São Paulo, São Paulo, Brazil).

2) Blood sampled at the beginning of the feedlot period on day 0.

3) Blood sampled at slaughter on day 103.
